# Single Ventricle Physiology May Not Preclude Cardiac Repair in the Setting of Congenital Diaphragmatic Hernia

**DOI:** 10.1177/21501351251386423

**Published:** 2026-03-03

**Authors:** Kylie I. Holden, Michael C. Scott, Ashley H. Ebanks, Amir M. Khan, D. Michael McMullan, Anthony Johnson, Charles S. Cox, Matthew T. Harting, Damien J. LaPar

**Affiliations:** 1 12340 McGovern Medical School at the University of Texas Health Science Center, Houston, TX, USA; 2 7274 Seattle Children's Hospital, Seattle, WA, USA

**Keywords:** congenital diaphragmatic hernia, congenital heart defects, single ventricle physiology, surgical risk stratification, mortality

## Abstract

**Objective:** Management for congenital heart disease (CHD) with congenital diaphragmatic hernia (CDH), particularly in single ventricle physiology, is challenging, with limited studies showing poor outcomes. This investigation evaluated outcomes for these complex patients. **Methods:** This retrospective cohort study (2007-2022) from the CDH Study Group analyzed mortality in patients with CDH and single ventricle physiology, using standard statistical methods and Cox regression for estimation. **Results:** A total of 9,261 CDH patients were identified, including 1,886 with CDH + CHD and 131 with single ventricle physiology. The median gestational age was 38 weeks and birthweight was 2.8 kg. Of 131 patients, 34 (26.0%) had chromosomal anomalies, 20/131 (15.3%) received extracorporeal life support (ECLS), 53 underwent diaphragm repair (A: 5/53 [9.4%], B: 16/53 [30%], C: 23/53 [43.4%], D: 9/53 {17%}), and 21 received cardiac palliation (STAT 2: 1/21 [4.8%], STAT 4: 8/21 [38.1%], STAT 5: 12/21 [57.1%]). Mortality was 71.0% (93/131), highest (74/77, 96.2%) in patients not receiving diaphragm or cardiac repair, and lowest (5/20, 25.5%) in those receiving both. Mortality increased with defect size, reaching 20.0% (1/5) for STAT 4 and 57.1% (4/7) for STAT 5 in type C defects, and 100.0% for STAT 4 in type D defects, with no type D patients undergoing STAT 5. Unadjusted analyses found Apgar scores, length of stay, diaphragmatic repair, CDHSG stage, and cardiac palliation (all *P* < .05) associated with mortality, while Cox regression identified ECLS (hazard ratio [HR] = 10.83) and chromosomal anomalies (HR = 19.08) as significant mortality factors. **Conclusions:** This study underscores the variability in mortality outcomes for patients with single ventricle physiology and CDH, emphasizing the crucial role of surgical management and identifying key mortality predictors to optimize patient outcomes.

## Background

Congenital diaphragmatic hernia (CDH) results from abnormal embryologic development of the diaphragm, leading to the herniation of abdominal organs into the hemithorax. This causes compression of the cardiopulmonary structures, resulting in pulmonary hypoplasia, pulmonary hypertension, and cardiac dysfunction.^1^ The severity of cardiopulmonary compromise can be stratified based on the anatomy of the defect, including its size and location. Larger defects are associated with more severe pulmonary hypertension, greater pulmonary hypoplasia, and increased cardiac dysfunction.^2–4^ While management of isolated CDH has improved over time, the presence of concomitant anomalies, specifically congenital heart disease (CHD), adds a significant layer of complexity to the care of these infants.^5^

The combination of CHD and CDH has been well-documented as having a poor prognosis, with increased mortality, significantly affecting prenatal counseling.^5,6^ However, the wide spectrum of CHD complicates the ability to generalize and standardize approaches and outcomes in this unique population. While several studies have offered varying insights into the morbidity and mortality of this patient population,^5–8^ few have specifically focused on the outcomes of patients with single ventricle physiology. Limited, underpowered evidence has consistently reflected poor outcomes.

Graziano et al first highlighted the poor prognosis of single ventricle physiology in the context of CDH, reporting an alarmingly low survival rate of just 5.1%.^9^ Building on this, Shiono et al specifically examined the outcomes of patients with CDH and single ventricle physiology who underwent surgical palliation, finding mixed results.^10^ While some showed improvements in survival and cardiac function, the overall survival remained dismal, with only a few patients surviving long enough to receive palliative heart surgery.^10^ This trend was further reinforced by Stewart et al, who also reported worse outcomes in patients with single ventricle physiology and concurrent CDH, solidifying the bleak outlook for this patient population.^^6^^

Overall, recent data on this diagnosis remain very limited, particularly regarding how outcomes could be stratified based on the severity of CDH. This study was designed to assess the outcomes for patients with single ventricle physiology stratified by the Congenital Diaphragmatic Hernia Study Group (CDHSG) stage.

## Methods

### Study Design and Registry

This was a retrospective study from the CDHSG registry. The CDHSG registry, established in 1995, included over 90 active centers focused on investigating key clinical questions related to CDH through a prospective, multicenter approach.^11–13^ Data from the CDHSG registry (Versions 3-5) for births between 2007 and 2022 were included, as 2007 marked the start of consistent collection of CDH diaphragm defect sizes (CDHSG stage).^4^ The use of the CDHSG Registry and performance of this study were approved by the University of Texas at Houston Center for the Protection of Human Subjects/Institutional Review Board (#HSC-MS-03-223; Ref #118886), with informed written consent for the publication of the study data waived under the IRB.

### Study Data Elements and Study Population

Patients with CDH and single ventricle anatomy resulting in single ventricle physiology—defined by diagnoses such as hypoplastic left heart syndrome, tricuspid atresia, double-inlet left ventricle, unbalanced atrioventricular septal defect, pulmonary atresia with hypoplastic right ventricle, double outlet right ventricle with hypoplastic left ventricle, and mitral atresia—were included. Patients without single ventricle physiology were excluded. Patient demographics, anatomic characteristics such as the presence of a chromosomal anomaly (defined as any deviation from the normal number or structure of chromosomes), operative details such as the need for long-term durable feeding access (defined as either the need for a gastrostomy tube or gastrojejunostomy tube), and outcomes were analyzed. In addition, CDH severity was classified using the CDHSG staging system, where Stage A and B defects were considered “low-risk” and Stage C and D defects were considered “high-risk,” based on defect size assigned by the surgeon during repair. Similarly, CHD defects were analyzed according to 17 investigator-defined anatomic/physiologic categories. Congenital heart disease cardiac operations were assigned to each diagnosis based on the Society of Thoracic Surgeons-European Association for Cardio-Thoracic Surgery Congenital Heart Surgery (STAT) categories 1-5, with STAT 1 operations associated with the lowest mortality risk and STAT 5 operations associated with the highest mortality risk (Supplemental 1).^14^ All classifications were reviewed by a team of pediatric surgery (MTH) and congenital cardiac surgery (DJL) experts for accuracy. Operation timing was based on patient age in days of life, with the difference (Δ time) between CDH and CHD operations calculated by subtracting the age at CHD operation from the age at CDH repair; in rare cases, CHD operations occurred prior to CDH repair, resulting in a negative Δ time.

### Outcomes

The primary outcome was to evaluate outcomes in this highly specific patient population. Secondary outcomes included associations with extracorporeal life support (ECLS), the presence of chromosomal anomalies, and the timing of both diaphragmatic and cardiac surgical interventions.

### Data Analysis

Data analysis was conducted using standard statistical methods. Categorical variables were presented as percentages, while continuous variables were reported as median (interquartile range [IQR]). Continuous variables such as estimated gestational age (EGA) and birthweight were categorized, with low birthweight defined as <2.5 kg and preterm birth defined as delivery <37 weeks gestation. Nonparametric tests, including the Mann-Whitney *U*, Kruskal-Wallis, and χ^2^ tests, were used to compare categorical and continuous data. Cox regression models were used to estimate mortality, with factors such as CDHSG defect size, birthweight, 1- and 5-min Apgar scores, the use of ECLS, and undergoing cardiac palliation included in the model.^2,4,15,16^ Each variable included in the model was selected based on prior evidence, univariate comparisons, and clinical expertise. Estimated measures of association between modeled factors and mortality were displayed as odds ratios with 95% confidence intervals. Relationships of statistical significance were determined by a *P* < .05. All statistical analyses were performed using StataIC v16.1 (StataCorp.). All figures were created using Prism 9 (GraphPad Software).

## Results

### Patient Characteristics

**Figure 1.**
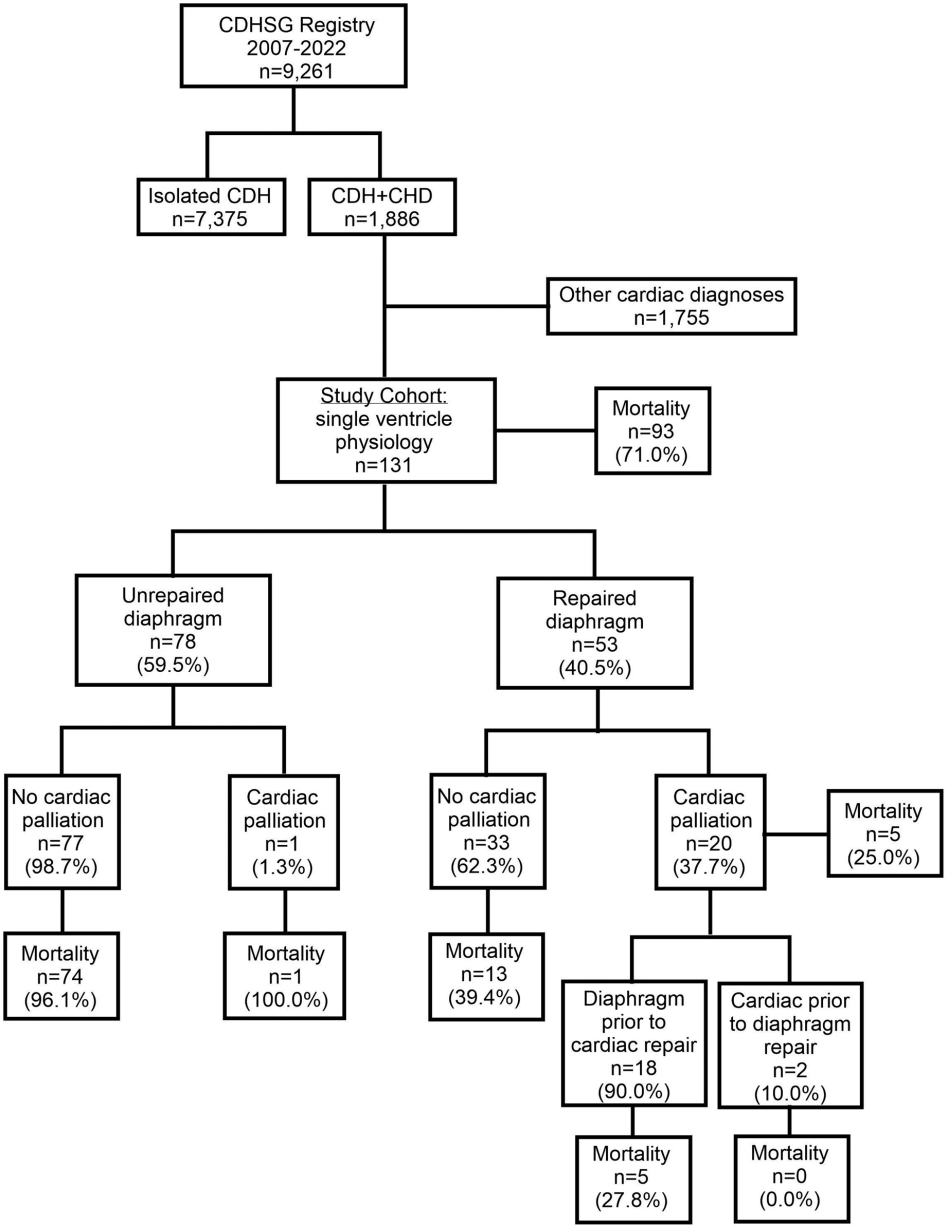
Population consort diagram. Abbreviations: CDH, congenital diaphrgmatic hernia; CDHSG, Congenital Diaphragmatic Hernia Study Group; CHD, congenital heart disease.

Overall, 9,261 CDH patients were identified, including 1,886 CDH + CHD patients and 131 patients who had single ventricle physiology. Overall mortality was 71.0% (n = 93/131); however, mortality rates varied depending on the nature of surgical intervention. Of the 131 patients, 78 did not undergo diaphragm repair; 77 of these also received no cardiac palliation, and one underwent a STAT 2 patent ductus arteriosus ligation but died shortly after. Mortality in this subgroup was 96.2% (74/77). Among the 53 patients who underwent diaphragm repair, 33 did not proceed to cardiac palliation, with a mortality of 39.4% (13/33) (Supplemental 2). It is important to note that the CDHSG captures only the initial hospitalization, so patients discharged may have been transferred for palliative care or returned later for cardiac intervention. The remaining 20 patients underwent sequential diaphragm and cardiac repair (Figure 1).

Table 1 displays the baseline characteristics of the study group of patients with single ventricle physiology (n = 131). The median gestational age was 38 weeks (IQR 37-39), with 27/131 (20.9%) born preterm (<37 weeks). Median birthweight was 2.8 kg (IQR 2.3-3.1), with 29.5% (11/38) classified as low birthweight (<2.5 kg). The median length of stay was 5 (1-53) days and the median time until death was 1 (0-13) days. Additionally, 34/131 patients (26.0%) had an associated chromosomal anomaly, but only 3/131 (8.8%) underwent both diaphragm and cardiac repair. Of the 20/131 patients (15.3%) who required ECLS, just 3/20 (15.0%) proceeded to sequential surgical intervention.

**Table 1.** Baseline Characteristics of All Patients With Single Ventricle Physiology (n = 131).

Patient characteristics				Missing values, n (%)
Estimated gestational age, median (IQR)		38	(37-39)	2 (1.5)
Preterm delivery (<37 weeks), n (%)		27	(20.9)	2 (1.5)
Birthweight (kg), median (IQR)		2.77	(2.3-3.1)	2 (1.5)
Low birthweight (<2.5 g), n (%)		38	(29.5)	2 (1.5)
1-min Apgar score, median (IQR)		3	(1-5)	8 (6.1)
5-min Apgar score, median (IQR)		5	(3-7)	8 (6.1)
Chromosomal anomalies, n (%)		34	(26.0)	1 (0.8)
Received extracorporeal life support (ECLS), n (%)		20	(15.3)	0 (0)
Underwent diaphragm repair, n (%)		53	(40.5)	0 (0)
CDHSG stage, n (%)	A	5	(9.4)	0 (0)
	B	16	(31.2)	
	C	23	(43.4)	
	D	9	(17.0)	
Left-sided defect, n (%)		103	(78.6)	1 (0.8)
Time to CDH repair, days, median (IQR)		6	(4-11)	0 (0)
Underwent cardiac repair, n (%)		21	(16.0)	0 (0)
STAT categories, n (%)	1	0	(0)	0 (0)
	2	1	(4.8)	
	3	0	(0)	
	4	8	(38.1)	
	5	12	(57.1)	
Time to CHD operation, days, median (IQR)		67	(33-92)	1 (4.8)
Underwent concomitant repair, n (%)		20	(15.3)	0 (0)
Time between operations, median (IQR)		14.5	(8-37)	0 (0)
Overall mortality, n (%)		93	(71.0)	0 (0)
Surgical feeding access, n (%)		15	(11.4)	1 (2.6)
Length of hospitalization, median (IQR)		5	(1-53)	0 (0)

Abbreviations: CDH, congenital diaphragmatic hernia; CDHSG, Congenital Diaphragmatic Study Group; CHD, congenital heart disease; IQR, interquartile range.

Among the 53 patients with single ventricle physiology who underwent diaphragm repair, the distribution of CDHSG defect size included: A (5/53, 9.4%), B (16/53, 31.2%), C (23/53, 43.4%), and D (9/53, 17.0%). Of those 53, 20 proceeded with sequential cardiac palliation with the distribution of STAT categories: STAT 2 (1/20, 4.8%), STAT 4 (8/20, 38.1%), and STAT 5 (12/20, 57.1%). While most patients underwent diaphragm repair prior to cardiac intervention, the two patients who received cardiac palliation before diaphragm repair both survived to discharge. Notably, neither of these patients had chromosomal abnormalities nor did they receive ECLS. One patient had a type A defect, while the other had a type C defect (Table 2).

**Table 2.** The Outcomes of Patients With Single Ventricle Physiology Who Underwent Sequential Diaphragm and Cardiac Repair (n = 20).^a^

Diagnosis	Surgery completed	STAT	CDHSG stage	Chromosomal anomaly	ECLS used	Outcome
Tricuspid atresia	Systemic-PA shunt	4	D	No	Yes	Survived
HLHS	Hybrid Norwood	5	C	No	No	Survived
HLHS	Norwood	5	B	No	No	Survived
HLHS	Norwood	5	A	No	No	Survived
Tricuspid atresia	PA band placement	4	A	No	No	Survived
HLHS	Norwood	5	C	Yes	No	Mortality
HLHS	Norwood	5	A	No	No	Survived
Tricuspid atresia	PA band placement	4	C	No	No	Survived
HLHS	Norwood	5	C	No	No	Survived
Functional SV/Hypoplastic LV/VSD	Arch reconstruction + PA band placement	4	C	No	No	Mortality
HLHS	Norwood	5	C	No	Yes	Mortality
HLHS	Norwood	5	C	No	No	Mortality
HLHS	Norwood	5	C	No	No	Survived
Tricuspid atresia	Systemic-PA shunt	4	C	No	No	Survived
Tricuspid atresia	PA band placement	4	C	Yes	No	Survived
HLHS	Norwood	5	C	No	Yes	Mortality
Tricuspid atresia	Systemic-PA shunt	4	A	No	No	Survived
HLHS	Norwood	5	B	No	No	Survived
HLHS	Norwood	5	B	No	No	Survived
Tricuspid atresia	Systemic-PA shunt	4	C	Yes	No	Survived

Abbreviations: CDHSG, Congenital Diaphramatic Hernia Study Group; ECLS, extracorporeal life support; HLHS, hypoplastic left heart syndrome; LV, left ventricle; PA, pulmonary artery; SV, single ventricle; VSD, ventricular septal defect.

a For each patient, the type of surgery completed, the STAT Classification, CDHSG stage, presence of chromosomal anomalies, use of ECLS, and the final outcome (survived or mortality) are listed. Data provide insight into the correlation between surgical approach, patient characteristics, and survival outcomes.

### Univariate Associations With Mortality

Table 3 shows univariate, unadjusted associations between various factors and mortality in all patients with single ventricle physiology (n = 131). Among the *total* population, 38 patients (29.0%) survived and 93 (71.0%) died. Mortality was associated with lower 1- and 5-min Apgar scores, shorter lengths of stay, absence of both isolated diaphragmatic and cardiac repair, and a lower likelihood of undergoing sequential surgical intervention.

**Table 3.** Unadjusted Comparisons of All Patients With Single Ventricle Physiology (n = 131) Compared Among Survivors and Decedents.

Univariate analysis				
Variable		Survival (n = 38)	Mortality (n = 93)	*P*
Birthweight (kg), median (IQR)		2.9 (2.6-3.1)	2.7 (2.1-3.3)	.21
Estimated gestational age (weeks), median (IQR)		38 (37-39)	38 (37-39)	.97
1-min Apgar score, median (IQR)		5 (3-7)	2 (1-5)	.**001**
5-min Apgar score, median (IQR)		7 (5-8)	4 (207)	**<**.**001**
Utilized ECLS, n (%)		4 (10.5)	16 (17.2)	.33
Chromosomal anomalies, n (%)		7 (18.4)	27 (29.0)	.35
Surgical feeding access, n (%)		14 (36.8)	1 (1.1)	.79
Length of hospitalization, median (IQR)		84.5 (43-108)	1 (0-13)	**<**.**001**
Underwent diaphragm repair, n (%)		35 (92.1)	18 (19.4)	**<**.**001**
Defect size, n (%)	A	5 (14.3)	0 (0.0)	.**002**
	B	15 (42.8)	1 (5.5)	
	C	12 (34.3)	11 (61.1)	
	D	3 (8.6)	6 (33.4)	
Left-sided defect, n (%)		30 (78.9)	73 (78.5)	.39
Time to CDH repair, days, median (IQR)		6 (4-11)	6 (3-10)	.59
Underwent cardiac repair, n (%)		15 (39.5)	6 (6.5)	**<**.**001**
STAT categories, n (%)	1	0 (0.0)	0 (0.0)	.16
	2	0 (0.0)	1 (16.7)	
	3	0 (0.0)	0 (0.0)	
	4	7 (46.7)	1 (16.7)	
	5	8 (53.3)	4 (66.6)	
Time to CHD operation, days, median (IQR)		76 (33-96)	51 (27-67)	.33
Underwent sequential repair, n (%)		15 (39.5)	5 (5.4)	**<**.**001**
Time between operations, median (IQR)		14 (4-42)	16 (11-20)	.75

Abbreviations: CDH, congenital diaphragmatic hernia; CDHSG, Congenital Diaphragmatic Hernia Study Group; CHD, congenital heart disease; ECLS, extracorporeal life support; IQR, interquartile range.

Bold implies statistical significance.

### Mortality by Congenital Diaphragmatic Hernia Stage Group

Table 4 shows the percent mortality by CDHSG stage and STAT category for patients with single ventricle physiology and CDH who underwent sequential diaphragm and cardiac repair. For patients with low-risk type A or B defects, no fatalities were observed in either the STAT 4 or STAT 5 categories. However, as the defect size increased to larger, high-risk types, mortality rates began to rise. Among those with a type C defect, the mortality rates were 20.0% (1/5) for STAT 4 operations and 57.1% (4/7) for STAT 5 operations. Notably, no patients with a type D defect received a STAT 5 operation.

**Table 4.** Percent Mortality (Number of Deaths/Total Patients) by CDHSG Defect Stage and STAT Category Among Patients Who Underwent Sequential Diaphragmatic and Cardiac Repair (n = 20).

		CDHSG stage			
		A	B	C	D
STAT category	4	0.0% (0/2)	NA (0/0)	20.0% (1/5)	0.0% (0/1)
	5	0.0% (0/2)	0.0% (0/3)	57.1% (4/7)	NA (0/0)

### Cox Regression Analysis

In a Cox regression analysis assessing mortality risk, the use of ECLS and the presence of chromosomal anomalies were found to be significantly associated with increased mortality, with hazard ratios (HRs) of 10.83 (*P* = .005) and 19.08 (*P* = .004), respectively, highlighting their impact on survival outcomes. Additional factors, including birthweight (HR = 0.2353, *P* = .057) and 5-min Apgar scores (HR = 0.6606, *P* = .077), showed marginal significance with reduced mortality risk. In contrast, EGA (HR = 1.2845, *P* = .404), CDHSG stage (HR = 1.3285, *P* = .581), 1-min Apgar scores (HR = 1.1929, *P* = .511), and undergoing cardiac palliation (HR = 0.3379, *P* = .212) did not demonstrate significant associations with mortality.

### Kaplan-Meier Survival Curve Analysis

**Figure 2.**
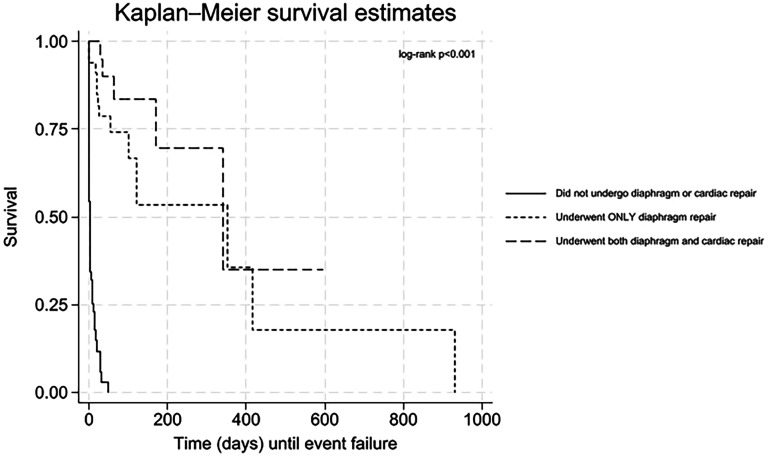
Kaplan-Meier survival curve comparing survival outcomes across three groups: (1) no diaphragm or cardiac repair, (2) diaphragm repair only, and (3) both diaphragm and cardiac repair.

Figure 2 shows the probability of survival over time for those who do not undergo either diaphragm or cardiac repair, for those that undergo just diaphragm repair, and for those that undergo sequential repair. The log-rank test yielded a χ^2^ statistic of 92.49 (*P* < .001), indicating a significant difference in survival between the groups.

### Unadjusted Associations Stratified by Subgroups

Patients born prematurely with low birthweight, chromosomal anomalies, and/or requiring ECLS often face significantly different postnatal trajectories, with wide variability in surgical options and expected outcomes. Table 5 presents the distribution of various patient subgroups based on birth status, birthweight, chromosomal anomalies, and the use of ECLS, across different surgical interventions. This table further stratifies the total population by patients who underwent neither diaphragm nor cardiac repair, those who had cardiac repair only, those who had diaphragm repair only, and those who received both diaphragm and cardiac repair. This breakdown provides a comprehensive clinical view, highlighting the frequency of prematurity, low birthweight, chromosomal anomalies, and ECLS use within each subgroup, as well as the associated mortality for each clinical profile. Most infants with single ventricle physiology were born at term, with normal birthweigts, no chromosomal anomalies, and did not utilize ECLS. When these patients underwent both surgical procedures, all survived to discharge. In contrast, patients born preterm with low birthweights, even without chromosomal anomalies or ECLS utilization, demonstrated a significantly lower survival rate, with 90.9% dying prior to discharge.

**Table 5.** More Precise Outcome Prediction by Stratifying Patients Based on Key Clinical Variables, Including Birthweight, Gestational Age, Presence of Chromosomal Anomalies, Use of ECLS, and Type of Surgical Intervention.^a^

	Total population n = 131	Unrepaired diaphragm and cardiac defect n = 77	Repaired cardiac defect only n = 1	Repaired diaphragm only n = 33	Repaired both diaphragm and cardiac defect n = 20
Term + normal birthweight + no chromosomal anomaly + no ECLS use	52/131 (61.5)	30/77 (100.0)	NA	13/33 (15.4)	9/20 (0.0)
Term + normal birthweight + chromosomal anomaly + no ECLS use	13/131 (84.6)	9/77 (88.9)	1/1 (100.0)	1/33 (100.0)	2/20 (50.0)
Term + normal birthweight + no chromosomal anomaly + ECLS use	13/131 (69.2)	3/77 (100.0)	NA	8/33 (62.5)	2/20 (50.0)
Term + normal birthweight + chromosomal anomaly + ECLS use	1/131 (100.0)	1/77 (100.0)	NA	NA	NA
Term + low birthweight + no chromosomal anomaly + no ECLS use	10/131 (70.0)	6/77 (83.3)	NA	1/33 (100.0)	3/20 (33.3)
Term + low birthweight + chromosomal anomaly + no ECLS use	8/131 (75.0)	5/77 (100.0)	NA	2/33 (50.0)	1/20 (0.0)
Term + low birthweight + no chromosomal anomaly + ECLS use	2/131 (100.0)	1/77 (100.0)	NA	NA	1/20 (100.0)
Term + low birthweight + chromosomal anomaly + ECLS use	1/131 (100.0)	1/77 (100.0)	NA	NA	NA
Preterm + normal birthweight + no chromosomal anomaly + no ECLS use	6/131 (50.0)	1/77 (100.0)	NA	4/33 (50.0)	1/20 (0.0)
Preterm + normal birthweight + chromosomal anomaly + no ECLS use	3/131 (33.3)	2/77 (50.0)	NA	1/33 (0.0)	NA
Preterm + normal birthweight + no chromosomal anomaly + ECLS use	0/131 (NA)	NA	NA	NA	NA
Preterm + normal birthweight + chromosomal anomaly + ECLS use	2/131 (100.0)	1/77 (100.0)	NA	1/33 (100.0)	NA
Preterm + low birthweight + no chromosomal anomaly + no ECLS use	11/131 (90.9)	9/77 (100.0)	NA	1/33 (0.0)	1/20 (100.0)
Preterm + low birthweight + chromosomal anomaly + no ECLS use	5/131 (80.0)	4/77 (100.0)	NA	1/33 (0.0)	NA
Preterm + low birthweight + no chromosomal anomaly + ECLS use	0/131 (NA)	NA	NA	NA	NA
Preterm + low birthweight + chromosomal anomaly + ECLS use	0/131 (NA)	NA	NA	NA	NA

Abbreviations: CDH, congenital diaphragmatic hernia; ECLS, extracorporeal life support.

a It represents the frequency of each subgroup and the corresponding mortality percentage of those who fit each specific subclassification (in parentheses) among patients with CDH and single ventricle physiology (n = 131), stratified by birth status (term vs preterm), birthweight (normal vs low), presence of chromosomal anomalies, and use of ECLS. Data are further broken down across clinically relevant subgroups: unrepaired diaphragm and cardiac defect (n = 77), cardiac repair only (n = 1), diaphragm repair only (n = 33), and those who underwent both diaphragm and cardiac repair (n = 20). “Mortality” refers to in-hospital deaths during the initial admission captured in the CDHSG registry.

## Discussion

Select infants with CDH have additional, associated anomalies, with cardiac defects occurring in 10% to 35% of cases.^9^ This represents a significant portion of the CDH population, and clinicians continue to explore ways to improve their care. Despite considerable efforts to better understand this population, there remains a notable gap in the literature focused on CDH patients with concomitant, specific cardiac diagnoses, particularly single ventricle physiology. Key studies, touch upon this complex subpopulation,^6,9,10,17^ however, the present study was specifically designed to deepen our understanding of this population, providing a more focused, contemporary analysis of a large sample size.

Focusing on this specific population was crucial, as they have historically been linked to a very poor prognosis. However, the present study challenges this traditional view and approach to managing these patients. A key factor in improving survival may be ensuring both diaphragmatic and cardiac repairs. These patients had previously been associated with poor outcomes due to the pulmonary parenchymal and arterial hypoplasia seen in CDH, which significantly affected the cardiovascular system and increased pulmonary vascular resistance.^18^ These cardiopulmonary changes were particularly impactful in cardiac patients who relied on adequately sized pulmonary arteries and normal pulmonary vascular resistance for optimal pulmonary blood flow. Patients with univentricular anatomy were especially vulnerable to the abnormal cardiopulmonary physiology associated with CDH.^9^ A critical aspect of their palliation involved eliminating the parallel circulation and establishing a circulation in series through cavopulmonary connections, which reduced volume overload on the single ventricle.^9^ These cavopulmonary connections relied on low pulmonary vascular resistance and normal pulmonary arterial size and morphology to ensure adequate pulmonary blood flow.^9^ While surgical intervention is vital for survival, the complex CDH physiology combined with single ventricle anatomy had historically been linked to poor outcomes, making these patients challenging candidates for cardiac surgery.^17^ The present study confirmed that mortality approached nearly 100% when surgery was not performed, but dropped to approximately 25% when surgical intervention was completed. Clearly, there is a selection bias here. Clinical teams, faced with extremely high-risk cases, carefully identify candidates based on multiple factors—often institution-specific and informed by limited available data—aiming to select those with a more favorable overall profile. One example, would be those with CDHSG Stage A diaphragm defects, which represented four patients in the present cohort and all of whom survived single ventricle palliation. A conservative, cautious approach appears to contribute to the observed reduction in mortality, highlighting both the progress made and the potential for broader inclusion in the future. To our knowledge, the present study is one of the most comprehensive and contemporary investigation of patients with CDH and single ventricle physiology, reporting lower mortality rates compared with the existing literature, particularly among those who underwent surgery.

Another adjunctive therapy that can be utilized in the management of these patients is ECLS. The use of ECLS was commonly linked to poor outcomes in patients with CDH + CHD. However, this was likely because ECLS was typically employed for the highest-risk patients, who often exhibit the most severe physiological compromise. Dyamenahalli et al argued that patients with CDH and CHD, including those with single ventricle physiology, who were supported by ECLS, had better predicted outcomes than previously believed.^19^ They reported a survival to discharge rate of approximately 55% for patients with CDH and single ventricle physiology.^19^ These findings were consistent with the present study, where 15.3% (n = 3/20) of patients with CDH and single ventricle physiology utilized ECLS, and 16 of these 20 patients survived, yielding a survival rate of 80.0%. In contrast, Stewart et al found no difference in the use of ECLS between those who survived and those who did not.^6^ However, this particular study stated that ECLS was not offered for patients with complex single ventricle physiology.^6^

### Limitations

The current study has limitations. Although the data were prospectively collected with the aim of addressing a predefined research question, the observational nature of the study led to a retrospective design, introducing the possibility of selection bias. Additionally, missing or incomplete data, although representing less than 1%, could not be incorporated into the analysis. Although the analyses were based on various predictive tools for CDH patients, there remains the potential for unmeasured confounding factors that may not have been fully accounted for in the multivariable models presented.^2^ Furthermore, the diversity of patient anatomy, and the diversity of care approaches, across the multicenter registry could have contributed to variations in echocardiographic interpretations, clinical management strategies, diagnoses of CHD, and the availability of specialized cardiovascular surgical support at different centers.^20^ There was a potential for imprecision in assignment of STAT categories due to the constraints of granularity of procedure-related data within the CDHSG clinical registry. The heterogeneity of the population, along with center-specific variations and surgeon-specific approaches, further underscore the need for individualized consideration when aiming for true surgical optimization. Additionally, the CDHSG database captures data only from the initial inpatient hospitalization through the time of discharge. This limitation may affect the accuracy of procedural and outcome data, particularly for patients who return for cardiac palliation during subsequent hospitalizations or are transferred to other institutions or home for palliative or hospice care. As a result, survival data may be skewed due to incomplete follow-up beyond the index hospitalization. Despite these limitations, this study provides valuable insights into critical gaps in the cardiac surgical literature, particularly regarding the impact of optimal surgical timing for CDH and CHD defects, based on a large, international, multi-institutional analysis.

## Conclusion

In conclusion, this study highlights the significant variability in survival outcomes among patients with single ventricle physiology and CDH, with surgical interventions playing a pivotal role in improving mortality rates. The data showed that patients who underwent both diaphragm repair and cardiac palliation experienced the lowest mortality, underscoring the importance of considering comprehensive surgical management for this complex patient population. Factors such as the use of ECLS and the presence of chromosomal anomalies were found to be strong predictors of mortality, further emphasizing the need for careful patient selection and individualized treatment plans. Additionally, the survival rates of preterm and low-birthweight infants were notably poorer, suggesting that these factors require consideration in clinical decision-making. Ultimately, this study contributed valuable insights into the management of CDH and single ventricle physiology, providing guidance for optimizing surgical interventions and improving outcomes for these complex, high-risk patients.

## Supplemental Material

sj-pdf-1-pch-10.1177_21501351251386423 - Supplemental material for Single Ventricle Physiology May Not Preclude Cardiac Repair in the Setting of Congenital Diaphragmatic Hernia Supplemental material, sj-pdf-1-pch-10.1177_21501351251386423 for Single Ventricle Physiology May Not Preclude Cardiac Repair in the Setting of Congenital Diaphragmatic Hernia by Kylie I. Holden, Michael C. Scott, Ashley H. Ebanks, Amir M. Khan, D. Michael McMullan, Anthony Johnson, Charles S. Cox, Matthew T. Harting and Damien J. LaPar in World Journal for Pediatric and Congenital Heart Surgery

sj-docx-2-pch-10.1177_21501351251386423 - Supplemental material for Single Ventricle Physiology May Not Preclude Cardiac Repair in the Setting of Congenital Diaphragmatic Hernia Supplemental material, sj-docx-2-pch-10.1177_21501351251386423 for Single Ventricle Physiology May Not Preclude Cardiac Repair in the Setting of Congenital Diaphragmatic Hernia by Kylie I. Holden, Michael C. Scott, Ashley H. Ebanks, Amir M. Khan, D. Michael McMullan, Anthony Johnson, Charles S. Cox, Matthew T. Harting and Damien J. LaPar in World Journal for Pediatric and Congenital Heart Surgery

## Abbreviations

CDH: congenital diaphragmatic hernia
CDHSG: Congenital Diaphragmatic Hernia Study Group
CHD: congenital heart disease
CI: confidence interval
DOL: days of life
ECLS: extracorporeal life support
HR: hazard ratio
IQR: interquartile range
OR: odds ratio
PDA: patent ductus arteriosus
STAT: Society of Thoracic Surgeons-European Association for Cardio-Thoracic Surgery Congenital Heart Surgery (Categories 1-5)
