# Supplementary material for: Single Ventricle Physiology May Not Preclude Cardiac Repair in the Setting of Congenital Diaphragmatic Hernia
Source: World J Pediatr Congenit Heart Surg. 2026 Mar 3;17(5):610–8. doi: 10.1177/21501351251386423 (PMC13443361; doi:10.1177/21501351251386423)
Supplement: Supplementary file 1 [file sj-pdf-1-pch-10.1177_21501351251386423.pdf]

Appendix C: The Society of Thoracic Surgeons - European Association for Cardio-Thoracic Surgery Congenital Heart Surgery Mortality Categories (STAT Mortality Categories) (June 24, 2020)

Procedure Harvest Code	Procedure Name	STAT Mortality Score	STAT Mortality Category
30	ASD repair, Patch	0.1	1
190	AVC (AVSD) repair, Partial (Incomplete) (PAVSD)	0.1	1
10	PFO, Primary closure	0.2	1
20	ASD repair, Primary closure	0.2	1
110	VSD repair, Patch	0.2	1
570	DCRV repair	0.2	1
780	Aortic stenosis, Subvalvar, Repair	0.2	1
1210	Coarctation repair, End to end	0.2	1
1360	Vascular ring repair	0.2	1
1470	ICD (AICD) implantation	0.2	1
1480	ICD (AICD) ([automatic] implantable cardioverter defibrillator) procedure	0.2	1
**2110	ASD Repair, Patch + PAPCV Repair	0.2	1
100	VSD repair, Primary closure	0.3	1
180	AVC (AVSD) repair, Intermediate (Transitional)	0.3	1
260	PAPVC repair	0.3	1
350	TOF repair, No ventriculotomy	0.3	1
360	TOF repair, Ventriculotomy, Nontransanular patch	0.3	1
580	Conduit reoperation	0.3	1
600	Valve replacement, Pulmonic (PVR)	0.3	1
680	Valve replacement, Aortic (AVR), Mechanical	0.3	1
690	Valve replacement, Aortic (AVR), Bioprosthetic	0.3	1
810	Sinus of Valsalva, Aneurysm repair	0.3	1
970	Fontan, TCPC, Lateral tunnel, Fenestrated	0.3	1
1250	Coarctation repair, Interposition graft	0.3	1
1460	Pacemaker procedure	0.3	1
1680	Glenn (Unidirectional cavopulmonary anastomosis) (Unidirectional Glenn)	0.3	1
*2120	PAPVC Repair, Baffle redirection to left atrium with systemic vein translocation (Warden) (SVC sewn to right atrial appendage)	0.3	1
520	1 1/2 ventricular repair	0.4	2
530	PA, Reconstruction (Plasty), Main (Trunk)	0.4	2
660	Valvuloplasty, Aortic	0.4	2
740	Ross procedure	0.4	2
820	LV to aorta tunnel repair	0.4	2
830	Valvuloplasty, Mitral	0.4	2
950	Fontan, Atrio-pulmonary connection	0.4	2
1330	PDA closure, Surgical	0.4	2
1365	Aortopexy	0.4	2
1450	Pacemaker implantation, Permanent	0.4	2
1500	Arrhythmia surgery - ventricular, Surgical Ablation	0.4	2
1690	Bilateral bidirectional cavopulmonary anastomosis (BBDCPA) (Bilateral bidirectional Glenn)	0.4	2
***2130	Superior Cavopulmonary anastomosis(es) + PA reconstruction	0.4	2
210	AP window repair	0.5	2

370	TOF repair, Ventriculotomy, Transanular patch	0.5	2
510	RVOT procedure	0.5	2
590	Valvuloplasty, Pulmonic	0.5	2
620	Conduit placement, LV to PA	0.5	2
715	Aortic root replacement, Bioprosthetic	0.5	2
720	Aortic root replacement, Mechanical	0.5	2
790	Aortic stenosis, Supravalvar, Repair	0.5	2
930	Pericardiectomy	0.5	2
1070	Congenitally corrected TGA repair, VSD closure	0.5	2
1220	Coarctation repair, End to end, Extended	0.5	2
1291	Anomalous origin of coronary artery from pulmonary artery repair	0.5	2
1380	Aortic aneurysm repair	0.5	2
1670	Bidirectional cavopulmonary anastomosis (BDCPA) (Bidirectional Glenn)	0.5	2
1730	Aneurysm, Ventricular, Left, Repair	0.5	2
1772	Conduit placement, Other	0.5	2
****2760	Hybrid Approach, Transcardiac balloon dilation	0.5	2
*2350	Explantation of pacing system	0.5	2
50	ASD, Common atrium (Single atrium), Septation	0.6	2
220	Pulmonary artery origin from ascending aorta (Hemitruncus) repair	0.6	2
270	PAPVC, Scimitar, Repair	0.6	2
735	Aortic root replacement, Valve sparing	0.6	2
840	Mitral stenosis, Supravalvar mitral ring repair	0.6	2
1000	Fontan, TCPC, External conduit, Fenestrated	0.6	2
1010	Fontan, TCPC, External conduit, Nonfenestrated	0.6	2
1290	Coronary artery fistula ligation	0.6	2
1790	Ligation, Pulmonary artery	0.6	2
****2770	Hybrid Approach, Transcardiac transcatheter device Placement	0.6	2
****2780	Fontan, TCPC, Intra/extracardiac conduit, Fenestrated	0.6	2
****2790	Fontan, TCPC, Intra/extracardiac conduit, Nonfenestrated	0.6	2
****3160	Kawashima operation (superior cavopulmonary connection in setting of interrupted IVC with azygous continuation)	0.6	2
****3180	Intravascular stent removal	0.6	2
*1305	Anomalous aortic origin of coronary artery from aorta (AAOCA) repair	0.6	2
*2100	Aortic stenosis, Subvalvar, Repair, With myectomy for IHSS	0.6	2
*2270	Valvuloplasty converted to valve replacement in the same operation, Pulmonic	0.6	2
*****3310	Fontan, TCPC, External conduit, hepatic veins to pulmonary artery, Fenestrated	0.6	2
*****3320	Fontan, TCPC, External conduit, hepatic veins to pulmonary artery, Nonfenestrated	0.6	2
85	Atrial fenestration closure	0.7	2
130	VSD, Multiple, Repair	0.7	2
250	Valve replacement, Truncal valve	0.7	2
290	Cor triatriatum repair	0.7	2
310	Atrial baffle procedure (Non-Mustard, Non-Senning)	0.7	2
340	Systemic venous stenosis repair	0.7	2
380	TOF repair, RV-PA conduit	0.7	2
460	Valvuloplasty, Tricuspid	0.7	2
470	Valve replacement, Tricuspid (TVR)	0.7	2
550	PA, Reconstruction (Plasty), Branch, Peripheral (At or beyond the hilar bifurcation)	0.7	2
910	Partial left ventriculectomy (LV volume reduction surgery) (Batista)	0.7	2

980	Fontan, TCPC, Lateral tunnel, Nonfenestrated	0.7	2
1230	Coarctation repair, Subclavian flap	0.7	2
1490	Arrhythmia surgery - atrial, Surgical Ablation	0.7	2
****3140	Hepatic vein to azygous vein connection, Direct	0.7	2
****3150	Hepatic vein to azygous vein connection, Interposition Graft	0.7	2
*2240	Valvuloplasty converted to valve replacement in the same operation, Aortic	0.7	2
*****3210	Removal of transcatheter delivered device from blood vessel	0.7	2
******3470	Coarctation repair, Extra-anatomic Bypass Graft	0.8	3
******3460	Coarctation repair, Desecending aorta anastomosed to ascending aorta	0.8	3
150	Ventricular septal fenestration	0.8	3
170	AVC (AVSD) repair, Complete (CAVSD)	0.8	3
240	Valvuloplasty, Truncal valve	0.8	3
330	Anomalous systemic venous connection repair	0.8	3
450	Occlusion MAPCA(s)	0.8	3
540	PA, reconstruction (plasty), Branch, Central (within the hilar bifurcation)	0.8	3
750	Konno procedure	0.8	3
1110	Arterial switch operation (ASO)	0.8	3
1240	Coarctation repair, Patch aortoplasty	0.8	3
1410	Transplant, Lung(s)	0.8	3
1630	Shunt, Ligation and takedown	0.8	3
1700	Hemifontan	0.8	3
1720	Aneurysm, Ventricular, Right, Repair	0.8	3
1740	Aneurysm, Pulmonary artery, Repair	0.8	3
**1275	Coarctation repair + VSD repair	0.8	3
*2280	Valvuloplasty converted to valve replacement in same operation, Tricuspid	0.8	3
*****3220	Removal of transcatheter delivered device from heart	0.8	3
70	ASD partial closure	0.9	3
960	Fontan, Atrio-ventricular connection	0.9	3
1150	Rastelli	0.9	3
1774	Conduit placement, Ventricle to aorta	0.9	3
1802	Pulmonary embolectomy, Acute pulmonary embolus	0.9	3
700	Valve replacement, Aortic (AVR), Homograft	1	3
*2290	Valvuloplasty converted to valve replacement in the same operation, Truncal valve	1	3
420	Pulmonary atresia - VSD (including TOF, PA) repair	1.1	3
1140	Mustard	1.1	3
1160	REV	1.1	3
1370	Pulmonary artery sling repair	1.1	3
610	Conduit placement, RV to PA	1.2	3
1800	Pulmonary embolectomy	1.2	3
*2310	Valvuloplasty converted to valve replacement in the same operation, Aortic - with Ross procedure	1.2	3
*2340	Fontan + Atrioventricular valvuloplasty	1.2	3
*****1145	Atrial baffle procedure, Mustard or Senning revision	1.2	3
******3350	PA reconstruction branch, peripheral, at or beyond the 1st lobar branch, proximal to first segmental branch	1.2	3
******3380	Extended ventricular septoplasty (modified Konno, VSD creation and patch enlargement of LVOT, sparing aortic valve) for tunnel type sub aortic stenosis	1.2	3
850	Valve replacement, Mitral (MVR)	1.3	4
920	Pericardial drainage procedure	1.3	4
****2750	Unifocalization MAPCA(s), Unilateral pulmonary Unifocalization	1.3	4

******3370	RV Rehabilitation, Endocardial resection	1.3	4
*2260	Valvuloplasty converted to valve replacement in the same operation, Mitral	1.3	4
*2300	Valvuloplasty, Common atrioventricular valve	1.3	4
890	Transplant, Heart	1.4	4
1025	Fontan revision or conversion (Re-do Fontan)	1.4	4
1180	DORV, Intraventricular tunnel repair	1.4	4
1200	DOLV repair	1.4	4
1280	Aortic arch repair	1.4	4
******3360	PA reconstruction branch, peripheral, (at or beyond 1st lobar branch, beyond first segmental branch	1.4	4
1650	PA debanding	1.4	4
1760	Cardiac tumor resection	1.4	4
**1120	Arterial switch operation (ASO) and VSD repair	1.4	4
**1123	Arterial switch procedure + Aortic arch repair	1.4	4
*2330	Superior cavopulmonary anastomosis(es) (Glenn or HemiFontan) + Atrioventricular valvuloplasty	1.4	4
400	TOF - Absent pulmonary valve repair	1.5	4
490	Valve excision, Tricuspid (Without replacement)	1.5	4
1300	Coronary artery bypass	1.5	4
1590	Shunt, Systemic to pulmonary, Modified Blalock-Taussig shunt (MBTS)	1.5	4
****2740	Unifocalization MAPCA(s), Bilateral pulmonary unifocalization - Incomplete unifocalization (not all usable MAPCA[s] are incorporated)	1.5	4
*****3200	PA band adjustment	1.5	4
390	TOF - AVC (AVSD) repair	1.6	4
465	Ebstein's repair	1.6	4
760	Ross-Konno procedure	1.6	4
1130	Senning	1.6	4
****2730	Unifocalization MAPCA(s), Bilateral pulmonary unifocalization - Complete unifocalization (all usable MAPCA[s] are incorporated)	1.6	4
****3130	Shunt, Systemic to pulmonary, Central (shunt from aorta), Central shunt with an end-to-side connection between the transected main pulmonary artery and the side of the ascending aorta (i.e. Mee shunt)	1.6	4
430	Pulmonary atresia - VSD - MAPCA repair	1.7	4
440	Unifocalization MAPCA(s)	1.7	4
730	Aortic root replacement, Homograft	1.7	4
1080	Congenitally corrected TGA repair, VSD closure and LV to PA conduit	1.7	4
1390	Aortic dissection repair	1.7	4
1640	PA banding (PAB)	1.7	4
****2710	Pulmonary atresia - VSD - MAPCA repair, Status post prior complete unifocalization (includes VSD closure + RV to PA connection [with or without conduit])	1.7	4
******3450	DORV-AVC (AVSD) repair	1.7	4
**1285	Aortic arch repair + VSD repair	1.7	4
140	VSD creation/enlargement	1.8	4
280	TAPVC repair	1.9	4
880	HLHS biventricular repair	1.9	4
*2230	Valve replacement, Common atrioventricular valve	1.9	4
*2250	Valvuloplasty converted to valve replacement in the same operation, Common atrioventricular	1.9	4
*2320	Valvuloplasty converted to valve replacement in the same operation, Aortic - with Ross-Konno procedure	1.9	4
******3390	LV Endocardial Fibroelastosis resection	1.9	4
300	Pulmonary venous stenosis repair	2	4
*****3230	Shunt, Systemic to pulmonary, Potts – Smith type (descending aorta to pulmonary artery)	2	4
1320	Interrupted aortic arch repair	2.1	4

1600	Shunt, Systemic to pulmonary, Central (From aorta or to main pulmonary artery)	2.1	4
****2720	Pulmonary atresia - VSD - MAPCA repair, Status post prior incomplete unifocalization (includes completion of pulmonary unifocalization + VSD closure + RV to PA connection [with or without conduit])	2.1	4
****2700	Pulmonary atresia - VSD - MAPCA repair, Complete single stage repair (1-stage that includes bilateral pulmonary unifocalization + VSD closure + RV to PA connection [with or without conduit])	2.3	4
230	Truncus arteriosus repair	2.4	4
**1125	Arterial switch procedure and VSD repair + Aortic arch repair	2.4	4
*2190	Aortic root translocation over left ventricle (Including Nikaidoh procedure)	2.4	4
*2210	TGA, Other procedures (Kawashima, LV-PA conduit, other)	2.4	4
60	ASD creation/enlargement	2.5	4
*2170	Hybrid Approach "Stage 1", Stent placement in arterial duct (PDA)	2.5	4
******3400	Double root translocation	2.6	4
80	Atrial septal fenestration	2.6	4
480	Valve closure, Tricuspid (Exclusion, Univentricular approach)	2.6	4
*2160	Hybrid Approach "Stage 1", Application of RPA and LPA bands	2.6	4
1660	Damus-Kaye-Stansel procedure (DKS) (Creation of AP anastomosis without arch reconstruction)	2.9	5
*2200	TAPVC repair + Shunt - Systemic to pulmonary	3	5
*2180	Hybrid Approach "Stage 1", Stent placement in arterial duct (PDA) + application of RPA and	3.1	5
900	Transplant, Heart and lung	3.2	5
1060	Congenitally corrected TGA repair, Atrial switch and Rastelli	3.2	5
1050	Congenitally corrected TGA repair, Atrial switch and ASO (Double switch)	3.4	5
****2755	Conduit insertion right ventricle to pulmonary artery + Intraventricular tunnel left ventricle to neoaorta + Arch reconstruction (Rastelli and Norwood type arch reconstruction) (Yasui)	3.6	5
*2150	Hybrid approach "Stage 2", Aortopulmonary amalgamation + Superior Cavopulmonary anastomosis(es) + PA Debanding + Without aortic arch repair	3.6	5
870	Norwood procedure	4	5
2140	Hybrid approach "Stage 2", Aortopulmonary amalgamation + Superior Cavopulmonary anastomosis(es) + PA Debanding + Aortic arch repair (Norwood [Stage 1] + Superior Cavopulmonary anastomosis(es) + PA Debanding)	4.1	5
**2220	Truncus + IAA Repair	5	5

*Indicates that this Procedure, Score, and Category were not included in the original JTCVS publication [6] but were subsequently assigned as part of the upgrade to version 3.0. The original list of procedure codes was based on Version 2.5 of the STS Congenital Heart Surgery Database. These additional procedures represent the list of new procedure codes that were added to The STS Congenital Heart Surgery Database in 2010 as part of the upgrade to version 3.0, and have also been incorporated into The EACTS Congenital Heart Surgery Database, and The Japan Congenital Cardiovascular Surgery Database (JCCVSD). To assign scores to these new procedures, a panel of highly experienced congenital heart surgeons from programs representing a variety of programmatic volume categories were surveyed and asked to provide an STS-EACTS Mortality Score for 26 procedures that were new to version 3.0, using the scores in the Table of the JTCVS article [6] as a guide. The mean of the scores from these ten surgeons was then used to assign the STS-EACTS Mortality Score and STS-EACTS Mortality Category for these 26 new procedures. (When the highest and lowest scores were discarded, the scores were essentially the same. [9/23 scores did not change, 13/23 scores change by only 0.1, and 1/23 scores change by 0.2]).

**Indicates a combined procedure (made up of two or more component procedures).

***Indicates a combined procedure and also a procedure for which the Score and Category were not part of the original JTCVS publication [6] and were assigned later as described above.

****Indicates that this Procedure, Score, and Category were not included in the original JTCVS publication [6] but were subsequently assigned as part of the upgrade to version 3.22. The original list of procedure codes was based on Version 2.5 of the STS Congenital Heart Surgery Database. These additional procedures represent the list of new procedure codes that were added to The STS Congenital Heart Surgery Database in 2014 as part of the upgrade to version 3.22, and have also been incorporated into The EACTS Congenital Heart Surgery Database, and The Japan Congenital Cardiovascular Surgery Database (JCCVSD). To assign scores to these new procedures, a panel of highly experienced congenital heart surgeons from programs representing a variety of programmatic volume categories were surveyed and asked to provide an STS-EACTS Mortality Score for 16 procedures that were new to version 3.22, using the STAT scores provided in the STS Congenital Heart Surgery Database Spring 2014 Feedback Report as a guide. The mean of the scores from these seventeen surgeons was then used to assign the STS-EACTS Mortality Score and STS-EACTS Mortality Category for these 16 new procedures. (When the high and low scores were discarded, the STAT Scores were essentially the same. [12/16 scores did not change and 4/16 scores change by only 0.1]; meanwhile, when the high and low scores were discarded, the STAT Categories were all unchanged.)

*****Indicates that this Procedure, Score, and Category were not included in the original JTCVS publication [6] but were subsequently assigned as part of the upgrade to version 3.3. The original list of procedure codes was based on Version 2.5 of the STS Congenital Heart Surgery Database. These additional procedures represent the list of new procedure codes that were added to The STS Congenital Heart Surgery Database in 2016 as part of the upgrade to version 3.3, and have also been incorporated into The EACTS Congenital Heart Surgery Database, and The Japan Congenital Cardiovascular Surgery Database (JCCVSD). To assign scores to these new procedures, a panel of highly experienced congenital heart surgeons from programs representing a variety of programmatic volume categories were surveyed and asked to provide an STS-EACTS Mortality Score for 7 procedures that were new to version 3.3, using the STAT scores provided in the STS Congenital Heart Surgery Database Spring 2016 Feedback Report as a guide. The mean of the scores from these seventeen surgeons was then used to assign the STS-EACTS Mortality Score and STS-EACTS Mortality Category for these 7 new procedures. (When the high and low scores were discarded, the STAT Scores were essentially the same.)

******Indicates that this Procedure, Score, and Category were not included in the original JTCVS publication [6] but were subsequently assigned as part of the upgrade to version 3.41. The original list of procedure codes was based on Version 2.5 of the STS Congenital Heart Surgery Database. These additional procedures represent the list of new procedure codes that were added to The STS Congenital Heart Surgery Database in 2019 as part of the upgrade to version 3.41, and have also been incorporated into The EACTS Congenital Heart Surgery Database, and The Japan Congenital Cardiovascular Surgery Database (JCCVSD). To assign scores to these new procedures, a panel of highly experienced congenital heart surgeons from programs representing a variety of programmatic volume categories were surveyed and asked to provide an STS-EACTS Mortality Score for 9 procedures that were new to version 3.41, using the STAT scores provided in the STS Congenital Heart Surgery Database Spring 2018 Feedback Report as a guide. The mean of the scores from these seventeen surgeons was then used to assign the STS-EACTS Mortality Score and STS-EACTS Mortality Category for these 9 new procedures. (When the high and low scores were discarded, the STAT Scores were essentially the same.)
