# Supplementary material for: Single Ventricle Physiology May Not Preclude Cardiac Repair in the Setting of Congenital Diaphragmatic Hernia
Source: World J Pediatr Congenit Heart Surg. 2026 Mar 3;17(5):610–8. doi: 10.1177/21501351251386423 (PMC13443361; doi:10.1177/21501351251386423)
Supplement: Supplementary file 2 [file sj-docx-2-pch-10.1177_21501351251386423.docx]

| Diagnosis | CDHSG Stage | Chromosomal Anomaly | ECLS Used | Outcome | Transfer vs Discharge |
| --- | --- | --- | --- | --- | --- |
| HLSH | B | No | No | Survived | DC |
| HLSH | B | Yes | No | Mortality | . |
| HLSH | D | No | Yes | Mortality | . |
| Ebstein's anomaly | C | No | Yes | Survived | TX |
| HLSH | C | No | No | Mortality | . |
| HLSH | C | No | No | Mortality | . |
| HLSH | B | No | No | Survived | DC |
| Ebstein's anomaly | B | No | No | Survived | DC |
| HLSH | B | No | No | Survived | DC |
| HLSH | D | Yes | No | Survived | TX |
| HLSH | D | No | Yes | Mortality | . |
| HLSH | D | Yes | No | Mortality | . |
| Ebstein's anomaly | C | No | No | Survived | DC |
| HLSH | A | No | No | Survived | DC |
| HLSH | B | No | No | Survived | DC |
| HLSH | B | No | Yes | Survived | DC |
| HLSH | C | No | Yes | Survived | DC |
| Ebstein's anomaly | B | No | No | Survived | DC |
| HLSH | C | No | Yes | Mortality | . |
| Tricuspid atresia | B | No | No | Survived | DC |
| Ebstein's anomaly | B | No | No | Survived | TX |
| HLSH | D | No | No | Mortality | . |
| Ebstein's anomaly | D | No | No | Survived | DC |
| Hypoplastic right ventricle | C | No | No | Survived | TX |
| HLHS | C | No | No | Mortality | . |
| Hypoplastic right ventricle | B | No | No | Survived | DC |
| HLHS | C | No | No | Mortality | . |
| HLHS | C | No | Yes | Mortality | . |
| HLHS | B | Yes | No | Survived | DC |
| Ebstein's anomaly | D | Yes | Yes | Mortality | . |
| Ebstein's anomaly | C | Yes | No | Survived | DC |
| HLHS | D | No | Yes | Mortality | . |
| Hypoplastic right ventricle | B | No | No | Survived | DC |

Supplemental 2:
This table summarizes the outcomes of patients with single ventricle physiology who underwent diaphragm but did NOT undergo cardiac repair (n=33). For each patient, their cardiac diagnosis is listed, CDHSG stage, presence of chromosomal anomalies, use of extracorporeal life support (ECLS), the final outcome (survived or mortality), and if they were discharged or transferred to another facility are listed.

Abbreviations: HLHS= hypoplastic left heart syndrome, TX= transfer, DC= discharge
